# Wilms tumor protein recognizes 5-carboxylcytosine within a specific DNA sequence

**DOI:** 10.1101/gad.250746.114

**Published:** 2014-10-15

**Authors:** Hideharu Hashimoto, Yusuf Olatunde Olanrewaju, Yu Zheng, Geoffrey G. Wilson, Xing Zhang, Xiaodong Cheng

**Affiliations:** 1 Department of Biochemistry, Emory University School of Medicine, Atlanta, Georgia 30322, USA;; 2 New England Biolabs, Ipswich, Massachusetts 01938, USA

**Keywords:** 5-carboxylcytosine, DNA modification, epigenetics

## Abstract

In mammalian DNA, cytosine occurs in several chemical forms, including unmodified cytosine (C), 5-methylcytosine (5mC), 5-hydroxymethylcytosine (5hmC), 5-formylcytosine (5fC), and 5-carboxylcytosine (5caC). 5mC is a major epigenetic signal that acts to regulate gene expression. 5hmC, 5fC, and 5caC are oxidized derivatives that might also act as distinct epigenetic signals. We investigated the response of the zinc finger DNA-binding domains of transcription factors early growth response protein 1 (Egr1) and Wilms tumor protein 1 (WT1) to different forms of modified cytosine within their recognition sequence, 5′-GCG(T/G)GGGCG-3′. Both displayed high affinity for the sequence when C or 5mC was present and much reduced affinity when 5hmC or 5fC was present, indicating that they differentiate primarily oxidized C from unoxidized C, rather than methylated C from unmethylated C. 5caC affected the two proteins differently, abolishing binding by Egr1 but not by WT1. We ascribe this difference to electrostatic interactions in the binding sites. In Egr1, a negatively charged glutamate conflicts with the negatively charged carboxylate of 5caC, whereas the corresponding glutamine of WT1 interacts with this group favorably. Our analyses shows that zinc finger proteins (and their splice variants) can respond in modulated ways to alternative modifications within their binding sequence.

## Abstract (precis)

Hashimoto et al. investigated the response of the zinc finger DNA-binding domains of transcription factors early growth response protein 1 (Egr1) and Wilms tumor protein 1 (WT1) to different forms of modified cytosine within their recognition sequence. 5-Carboxylcytosine (5caC) affected the two proteins differently, abolishing binding by Egr1 but not by WT1. In Egr1, a negatively charged glutamate conflicts with the negatively charged carboxylate of 5caC, whereas the corresponding glutamine of WT1 interacts with this group favorably.

In eukaryotic genomes, DNA methyltransferases convert a proportion of the cytosines (Cs), primarily in CpG dinucleotides, into 5-methylcytosine (5mC) (Bestor et al. 1988; Okano et al. 1998). Ten-eleven translocation (Tet) dioxygenases then convert a fraction of these to 5-hydroxymethylcytosine (5hmC), 5-formylcytosine (5fC), and 5-carboxylcytosine (5caC) in consecutive oxidation reactions (Kriaucionis and Heintz 2009; Tahiliani et al. 2009; Globisch et al. 2010; Ito et al. 2010, 2011; He et al. 2011). These modifications protrude into the major groove of DNA, the primary recognition surface for proteins, and change its atomic shape and pattern of electrostatic charge. In principle, such changes can alter the way in which proteins bind to their recognition sequences in DNA by strengthening the interactions, weakening them, or abolishing them altogether. This, in turn, can modulate gene expression and control cellular metabolism and is believed to be one of the principal mechanisms underlying epigenetic processes such as differentiation, development, aging, and disease.

Three well-characterized classes of mammalian proteins interact with DNA in a methylation-dependent manner. Methyl-binding domains (MBDs) recognize fully methylated CpG sequences in which both DNA strands contain 5mC (Baubec et al. 2013). SET and RING finger-associated (SRA) domains recognize hemimethylated CpG sequences containing 5mC in only one strand, such as arise during DNA replication (Bostick et al. 2007; Sharif et al. 2007). In addition, certain Cys2-His2 (C2H2) zinc finger (ZnF) proteins bind preferentially to longer, specific DNA sequences when internal CpG sites are methylated (Sasai et al. 2010; Liu et al. 2013b). The ability of ZnF proteins to respond to methylation in this way is significant because “sequences longer than CpG would be necessary for the regulation of gene expression by methylation” (Holliday 1996). The structures of three ZnF domains bound to 5mC-containing DNA were solved recently from the transcription factors Kaiso, Zfp57, and Klf4 (Buck-Koehntop et al. 2012; Liu et al. 2012, 2014). Here we investigate the binding of ZnF domains to oxidized modifications of 5mC.

In conventional C2H2 ZnF proteins, each finger comprises two β strands and one helix and generally interacts with three adjacent DNA base pairs (Wolfe et al. 2000; Klug 2010). Amino acid side chains from the N-terminal portion of the helix together with the preceding residue make major groove contacts with the bases of primarily one DNA strand. Almost always, the first zinc-binding histidine is positioned in the middle of the helix, separated from the preceding cysteine by 12 residues. In the discussion below, we use this histidine as reference position 0 and number the residues that make base contacts from this rather than from the more variable first position of the α helix. Most commonly, proceeding leftward in the amino acid sequence toward the N terminus, residues at positions −1, −4, and −7 (or −8) make base-specific contacts through their side chains; the identities of these amino acids are the principle determinants of the DNA sequence recognized (Supplemental Fig. S1A), although by no means the only ones (Gupta et al. 2014; Persikov and Singh 2014).

Zfp57 (with two ZnFs in tandem) and Klf4 (with three) recognize the triplet 5′-G-5mC-G-3′ within a 6-base-pair (bp) (Quenneville et al. 2011) and a 9-bp (Chen et al. 2008) sequence, respectively. In Zfp57 (Liu et al. 2012), arginine (R) at position −1 contacts the 5′ guanine (Gua), glutamate (E) at −4 interacts with the 5mC, and R at −8 contacts the 3′ Gua (Supplemental Fig. S1A). Similar interactions occur with Klf4 (Liu et al. 2014), except that the 3′ Gua is contacted by R at −7 rather than −8 (Supplemental Fig. S1A). Although predicting the binding of ZnFs to modified DNA remains a challenge, we found that four ZnFs from two other transcription factors, early growth response protein 1 (Egr1; also known as Zif268) and Wilms tumor protein 1 (WT1), also recognize DNA sequences containing 5′-GCG-3′ (Supplemental Fig. S1A). All four fingers have R at positions −1 and −7; three have E at −4, and one (in WT1) has glutamine (Q) at position −4 instead (Supplemental Fig. S1A). We investigated the behavior of Egr1 and WT1 toward sequences containing all forms of modified C and report our findings here.

## Results

Egr1/Zif268 belongs to a group of early response proteins whose genes are dramatically and rapidly induced upon stimulation by many environmental signals, including growth factors, hormones, and neurotransmitters (Pagel and Deindl 2011). The three-finger-binding domain of Egr1/Zif268 (Supplemental Fig. S1B) is one of the best-studied C2H2 ZnF proteins structurally (Pavletich and Pabo 1991). It binds to the consensus sequence 5′-GCG(T/G)GGGCG-3′ and has been used as a framework for engineering novel DNA-binding specificities (Wolfe et al. 2000; Klug 2010). This sequence contains two CpG sites for C modification that are dynamically modified in mouse embryonic stem cells (see the Supplemental Material). WT1, encoded by a complex gene characterized by many isoforms (Hohenstein and Hastie 2006; Ozdemir and Hohenstein 2014), contains four C2H2 ZnFs (Supplemental Fig. S1C). The first of these does not contact the bases (Stoll et al. 2007) and contributes little to specificity (Hamilton et al. 1995; Nakagama et al. 1995). Consequently, WT1 binds the same consensus sequence as Egr1/Zif268 (Stoll et al. 2007) and, in some cases, antagonizes Egr1/Zif268 function (Ritchie et al. 2010). For the work described here, we used a construct of WT1 containing only ZnF2 through ZnF4, which was structurally analogous to Egr1/Zif268. We compared the binding affinities and the crystal structures of the two protein domains with the same DNA sequences.

### 5mC substrates

**Figure 1.**
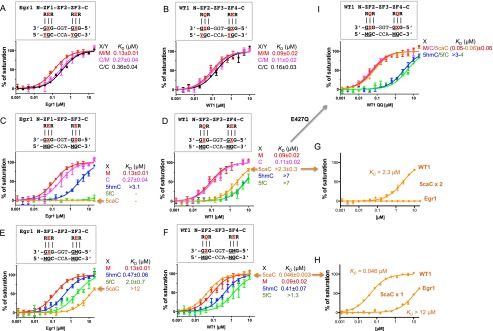
Egr1/Zif268 and WT1 bind methylated and 5mC oxidized DNA. Binding affinities of Egr1/Zif268 and WT1 with oligos containing varied forms of cytosine, as measured by fluorescence polarization assays. (*A*,*B*) Oligos fully methylated at both sites (M/M), unmethylated at both sites (C/C), or methylated in only the bottom strand at both sites (C/M). (*C*,*D*) Oligos modified in only the top strand at both sites with 5mC (M), C, 5hmC, 5fC, or 5caC. 5mC was present in the bottom strand at both sites in all cases. (*E*,*F*) Oligos modified in only the top strand at the 3′ site. All other sites contained 5mC (M). (*G*,*H*) Comparison of Egr1 and WT1 with oligos containing 5caC in the top strand at both sites (5caCx2) or only the 3′ site (5caCx1) using the data from *C*–*F*. (*I*) The “QQ” variant (Q369 and E427Q) of WT1 displays enhanced binding with 5caCx2 oligos.

We compared the binding of Egr1/Zif268 and WT1 with unmodified and 5mC-modified consensus sequences. Fluorescence polarization was used to measure the dissociation constant (*K*_D_) between the two binding domains and double-stranded oligonucleotides (oligos) that were either unmethylated (C/C) or fully methylated (M/M) at both internal CpG dinucleotide sites. Egr1/Zif268 and WT1 showed slightly higher affinity for the fully methylated sequence by factors of ∼2.8 (Egr1) and ∼1.8 (WT1) (Fig. 1A,B). With these ZnFs, only one DNA strand is involved in base-specific contacts (the “top” strand, the one depicted as the recognition sequence), while the other strand interacts mainly with water molecules (Supplemental Figs. S2, S3). We replaced the two 5mC bases in the top strand with unmodified C. Affinity for this hemimethylated (C/M) sequence dropped by factors of ∼2 (Egr1) and ∼1.2 (WT1) (Fig. 1A,B) to values intermediate between those of the completely methylated and the completely unmodified sequences. These results indicate that 5mC methylation of the CpG dinucleotides within the consensus sequence enhances the binding affinity of Egr1 and WT1 only modestly.

### Oxidized 5mC substrates

In turn, we replaced the two 5mC bases in the top strand with all three oxidized modifications (5hmC, 5fC, and 5caC) and repeated the binding assays. 5mC was present in the bottom strand in each case. For Egr1/Zif268, oxidation reduced binding significantly for 5hmC (by a factor of ∼24 compared with the M/M substrate) and abolished it completely for 5fC and 5caC (Fig. 1C). For WT1, oxidation reduced binding significantly but did not abolish it in any instance. 5hmC and 5fC reduced affinity by a factor of ∼75; 5caC reduced it less, by a factor of ∼25 (Fig. 1D). In contrast to Egr1, which cannot bind to 5fC or 5caC, WT1 retained substantial affinity for these modifications, particularly for 5caC (*K*_D_ > 2.3 μM) (Fig. 1D).

### Asymmetrically modified substrates

We next analyzed the effect of oxidized C at only the 3′ GCG triplet. 5mC was present in the bottom strand, as before, and also in both strands of the 5′ GCG triplet. Egr1 exhibited progressively weaker binding to the oxidized forms: 5hmC (3.6-fold lower than 5mC), 5fC (15-fold lower), and 5caC (>90-fold lower) (Fig. 1E). WT1 behaved similarly for 5hmC (4.5-fold lower than 5mC) and 5fC (14-fold lower) but, in striking contrast to Egr1, bound more strongly (twofold higher) to 5caC than to 5mC. The affinity of Egr1 for this site is thus 5mC > 5hmC > 5fC > 5caC, whereas for WT1, it is 5caC > 5mC > 5hmC > 5fC (Fig. 1F).

### Differential ZnF affinities

Comparing affinities for one-site and two-site modifications (Fig. 1G,H) indicates that the N-terminal ZnF2 of WT1 (which interacts with the 3′ GCG) has the highest affinity for 5caC (*K*_D_ ∼ 50 nM), while the C-terminal ZnF4 (which interacts with the 5′ GCG) and both of those of Egr1 have the lowest affinity for 5caC (*K*_D_ > 12 μM). This imbalance, we surmise, results in an intermediate affinity of *∼*2.3 μM for WT1 toward sequences containing 5caC at both positions (5caCx2 DNA). The principle difference between the two WT1 ZnFs is the residue at position −4, which is E in the low-affinity ZnF4 and Q in the high-affinity ZnF2. We used site-specific mutagenesis to change the E at position −4 of ZnF4 to Q (E427Q). The double “QQ” variant, as expected, now exhibited a higher affinity for the 5caCx2 sequence, although not markedly higher than for sequences containing C or 5mC (Fig. 1I). Affinity for DNA containing 5hmCx2 or 5fCx2 was little changed (Fig. 1I). In contrast to WT1 (QE) and the QQ mutant, Egr1 (the “EE” combination) has negligible affinity (no detectable binding) for 5caCx2 DNA (Fig. 1G).

### Structural investigations

**Figure 2.**
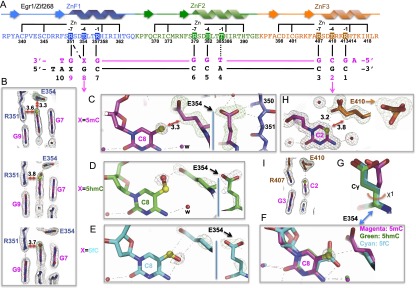
Egr1 binds methylated and unmodified DNA. (*A*) Schematic representation of the ZnF1–3 DNA-binding domain of Egr1/Zif268. The sequence and the secondary structure are shown. Arrows represent β strands, lines represent loops, and ribbons represent α helices. Two cysteine and two histidine residues (C2H2) in each finger are responsible for Zn^2+^ ligand binding (*top* connecting lines). Amino acids at positions −1, −4, and −7 (highlighted) relative to the first histidine interact specifically with the DNA bases shown *below*. The sequence of the oligos used for this study is shown with the top strand (magenta) oriented left to right from 3′ to 5′ with a 5′ overhanging adenine. The complementary strand (black) has a 5′ overhanging thymine. (*B*) Arg351 at position −7 of ZnF1 forms a methyl–Arg–Gua triad with the top strand of XpG (X = 5mC, 5hmC, or 5fC). The 2Fo − Fc electron density, contoured at 1σ above the mean, is shown in gray. (*C*) E354 at position −4 of ZnF1 is in van der Waals contact with the methyl group of 5mC (red arrow; distance shown in angstroms). The simulated annealing omit electron densities (meshed lines), contoured at 10σ and 4σ above the mean, respectively, for omitting the methyl group of 5mC and the side chain of E354 are shown. (*D*,*E*) The side chain of E354 becomes disordered with 5hmC (*D*) or 5fC (*E*). (*E*) An intrabase H-bond is present between the formyl oxygen of 5fC and the *N*4 group. Simulated annealing omit electron densities (meshed lines), contoured at 10σ and 4σ above the mean, respectively, for omitting the hydroxyl group of 5hmC (*D*) (or the carbonyl oxygen atom of 5fC [*E*)] and the side chain of E354 are shown. (*F*,*G*) Conformation of E354 indicates ∼90° side chain rotations with 5hmC (green) and 5fC (cyan) compared with 5mC (magenta). (*H*,*I*) E410 at position −4 of ZnF3 adopts two conformations with unmodified cytosine, engaging in van der Waals and weak C-H…O type H-bond interactions with the ring carbon atoms.

**Figure 3.**
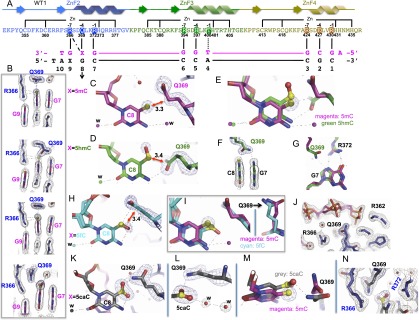
WT1 binds 5caC and 5mC. (*A*) Schematic of ZnF2–ZnF4 DNA-binding domain WT1 (−KTS isoform), depicted as in Figure 2. (*B*) The side chain of Q369 at position −4 of ZnF2 adopts different conformations with (from *top* to *bottom*) 5mC, 5hmC, 5fC, and 5caC. The 2Fo − Fc electron density, contoured at 1σ above the mean, is shown in gray. (*C*) Q369 is in van der Waals contact with the methyl group of 5mC (red arrow; distance in angstroms). The simulated annealing omit electron densities (meshed lines), contoured at 10σ and 4σ above the mean, respectively, for omitting the methyl group of 5mC and the side chain of Q369 are shown. (*D*) Q369 forms an H-bond (dotted line) with the *N*4 atom of 5hmC and is in van der Waals contact (red arrow) with the CH_2_ group. (*E*) Superimposition of *C* and *D* showing side chain conformation of Q369 with 5mC (magenta) and 5hmC (green). The two conformations are related by rotations of χ1 = 120°, χ2 = 90°, and χ3 = 90°. (*F*) Q369 interacts with 5hmC via the side chain carbonyl oxygen and with the 5′ Gua via the amide group. (*G*) R372 interaction with Gua7 in the presence of 5mC (magenta) and 5hmC (green). (*H*) Q369 interaction with 5fC. An intrabase H-bond is present in 5fC as in Egr1 (Fig. 2E). (*I*) Superimposition of *C* and *H* showing side chain conformation of Q369 with 5mC (magenta) and 5fC (cyan). (*J*) The three phosphate groups immediately surrounding 5fC are mobile. (*K*) One carboxylate oxygen of 5caC forms an H-bond with the side chain amide group of Q369. The other forms an intrabase H-bond with the *N*4 group. (*L*) Water-mediated interactions surrounding 5caC and Q369. The simulated annealing omit electron densities (meshed lines), contoured at 10σ and 4σ above the mean, respectively, for omitting the carboxylate group of 5caC and the side chain of Q369 are shown. (*M*) Superimposition of *C* and *K* showing Q369 with 5mC (magenta) and 5caC (gray). The two side chain conformations are related by a 70° rotation of the χ3 torsion angle. (*N*) Electrostatic sandwich between the negatively charged carboxylate group of 5caC and the positively charged guanidino groups R372 and R366.

To understand why Egr1 and WT1 respond so differently to 5caC, we determined the cocrystal structures of each protein with 10-bp oligos containing modified forms of the consensus sequence (Figs. 2, 3). We kept both strands of the 5′ GCG triplet in an unmodified state and modified both strands of the 3′ GCG triplet. We determined three structures for Egr1, with oligos containing 5mC, 5hmC, and 5fC in the resolution range of 1.6–2.1 Å, and four structures for WT1, with 5mC, 5hmC, 5fC, and 5caC in the resolution range of 1.5–2.1 Å (Supplemental Table S1). The three ZnFs of both proteins bind in the major DNA groove (Supplemental Figs. S2A, S3A). The C-terminal fingers (ZnF3 of Egr1 and ZnF4 of WT1) interact with the 5′ triplet (GCG), the middle fingers (Egr1-ZnF2 and WT1-ZnF3) interact with the central triplet (TGG), and the N-terminal fingers (Egr1-ZnF1 and WT1-ZnF2) interact with the 3′ triplet (modified GCG) (Supplemental Figs. S2, S3).

In Egr1, the methyl group of 5mC in the top strand makes van der Waals contact with the side chain guanidino group of R351 at position −7 of ZnF1 (Fig. 2A), which forms two hydrogen bonds (H-bonds) with the 3′ Gua (Fig. 2B), forming a methyl–Arg–Gua triad (Liu et al. 2013b). The methyl group also makes van der Waals contacts with the Cγ carbon atom of E354 (Fig. 2B,C), perpendicular to the methyl–Arg interaction. When the methyl group is oxidized to 5hmC or 5fC, the side chain of E354 loses rigidity, as indicated by the broken electron densities (Fig. 2D,E), which might contribute toward the decreased binding affinity.

Superimposing the 5mC, 5hmC, and 5fC structures revealed that E354 undergoes a significant change upon binding the oxidized modifications (Fig. 2F). Assuming that the broken density corresponds to the E354 side chain, the Cγ atom moves away from the 5-methyl carbon by rotations of side chain torsion angles (Fig. 2G). In the 5hmC structure, the hydroxyl oxygen points away from the *N*4 amino group of C and interacts with the side chain carboxyl of E354 (Fig. 2D), whereas in the 5fC structure, the formyl oxygen points in the other direction and forms an intrabase H-bond with the *N*4 amino group (Fig. 2E). The latter interaction results in the exclusion of a water molecule that is present in the 5mC and 5hmC structures but absent in the 5fC structure (Fig. 2C–E).

The interactions with unmodified C can be inferred from earlier cocrystal structures of Egr1/Zif268 (Supplemental Fig. S4; Pavletich and Pabo 1991) and from the C-terminal ZnF3 at the unmodified 5′ GCG site (Fig. 2A). E410 at position −4 of ZnF3 adopts two slightly different side chain conformations. Both appear to be in van der Waals contact with ring carbon-5 of the C, and one might also form a weak (3.2 Å) C-H…O type bond with carbon-5 or carbon-6 (Fig. 2H; Horowitz and Trievel 2012). The interaction corresponding to the methyl–Arg contact is absent, but some stacking between the C ring and the side chain of Arg at position −7 continues (Fig. 2I).

For WT1, the higher binding affinity for 5caC allowed us to determine the structure of the complex with this modification in addition to those with the others. R366 at position −7 of WT1 ZnF2, like R351 of Egr1, forms H-bonds with the 3′ Gua and van der Waals contacts with the 5mC, 5hmC, and 5fC groups (Fig. 3B). The side chain of Q369 at position −4 contacts the methyl group of 5mC with a conformation similar to that of E354 of Egr1 (Fig. 3C) but adopts a quite different conformation with 5hmC (Fig. 3D) and yet another conformation with 5caC. Movement of Q369 from the conformation that it adopts with 5mC to that with 5hmC requires rotations of all three side chain torsion angles (Fig. 3E). Rather than interacting with the 5hmC hydroxyl, Q369 in this different conformation appears to form two H-bonds with bases: one with the *N*4 amino group of 5hmC (via the side chain carbonyl oxygen) and the other with the N7 ring atom of the 5′ Gua (via the side chain amide group) (Fig. 3F). The latter H-bond weakens the interaction between Gua and R372 at position −1, pulling Gua closer and pushing R372 away (Fig. 3G). The water molecule mentioned previously is present in the 5mC structure but not in the 5hmC structure due to the different conformation of Q369 (Fig. 3C,D).

The Q369–5fC interaction in WT1 (Fig. 3H) is similar to the Q369–5mC interaction except for minor differences in side chain conformation (Fig. 3I). However, for reasons unclear to us, the three phosphate groups (two 5′ and one 3′) immediately surrounding 5fC can all be modeled with multiple conformations, indicating flexibility of the local DNA structure (Fig. 3J). This flexibility is not observed in the other WT1 structures (all of which crystallized in the same space group) or the Egr1 structures with the same oligos.

The 5caC bound by WT1 participates in intramolecular and intermolecular interactions. An intrabase H-bond is present between the *N*4 amino group and one of the 5caC carboxylate oxygens (Fig. 3K). The second carboxylate oxygen atom forms an H-bond with the amide of Q369 (Fig. 3K,L), the side chain of which has rotated ∼70° around the χ3 torsion angle from the conformation in which it interacts with 5mC (Fig. 3M). The negatively charged carboxylate group is also sandwiched between the positively charged guanidino groups of R372 and R366 that recognize the neighboring Guas (Fig. 3N). The 5caC carboxylate group is further stabilized by water-mediated interactions (Fig. 3L) that also involve the side chain of the adjacent amino acid, S365. The corresponding residue in ZnF4, A423, cannot interact with water in this way, and this might explain why the affinity of the double “QQ” mutant E427Q for DNA containing 5caC at both positions is no greater than it is for DNA containing C or 5mC instead (Fig. 1I).

### The WT1 +KTS (Lys–Thr–Ser) splice isoform binds most strongly to 5caC DNA

**Figure 4.**
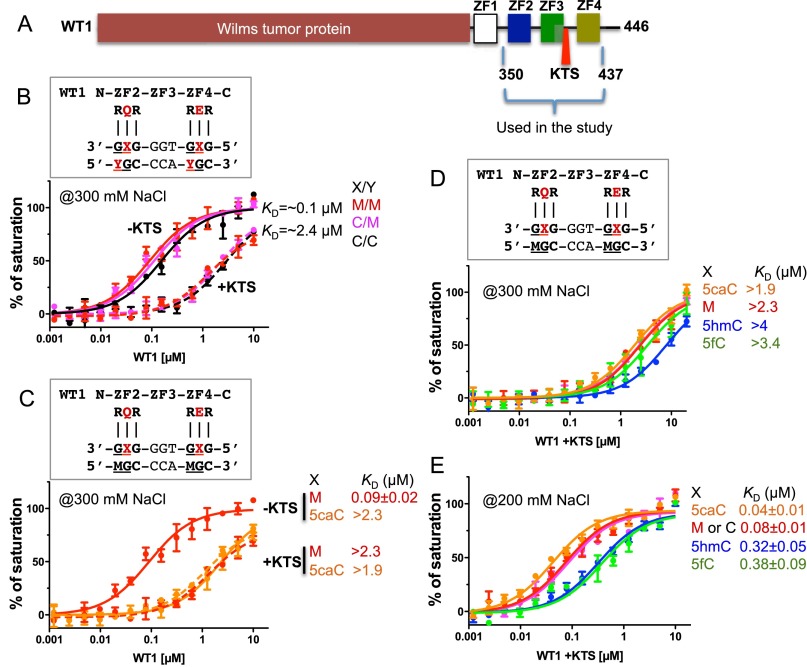
The WT1 +KTS isoform binds most strongly to 5caC DNA. (*A*) Human WT1 contains a C-terminal ZnF DNA-binding domain comprising four fingers in tandem. For the study described here, we used a fragment of WT1 containing ZnF2, ZnF3, and ZnF4 without KTS (the −KTS isoform) and with KTS (the +KTS isoform). (*B*) Comparison of the ±KTS isoforms on oligos containing unmodified C or 5mC (fully or hemimethylated). (*C*) The two KTS isoforms have a similar, relatively low affinity for 5caC-containing DNA but substantially different affinities for 5mC-containing DNA. (*D*,*E*) The +KTS isoform binds most strongly to 5caC-containing DNA. Affinity is uniformly low in 300 mM NaCl (*D*) but considerably higher in 200 mM NaCl (*E*).

WT1 is encoded by a complex gene characterized by many isoforms (Hohenstein and Hastie 2006; Ozdemir and Hohenstein 2014). All known isoforms of WT1 include four ZnFs at the C terminus with or without three extra amino acids (KTS) between ZnF3 and ZnF4 (Hohenstein and Hastie 2006; Ozdemir and Hohenstein 2014). Mutations in the splice site of WT1 that change the normal +KTS/−KTS ratio of 60:40 to 40:60 lead to Frasier syndrome (Barbaux et al. 1997). The preceding discussions pertain only to the −KTS isoform. We also expressed and purified the +KTS isoform in the context of ZnF2–ZnF4 and compared the binding affinities of the two isoforms with variously modified substrates (Fig. 4A). The +KTS isoform has greatly reduced binding affinity for oligos containing unmodified C or 5mC (fully or hemimethylated) compared with the −KTS isoform by a factor of ∼24 (Fig. 4B). This reduced affinity might result from increased linker flexibility due to the additional amino acids that leads to loss of binding by ZnF4 (Laity et al. 2000b). Both ±KTS isoforms have a similar, relatively low, affinity for 5caCx2-containing DNA but substantially different (25-fold) affinities for 5mC-containing DNA (Fig. 4C). This suggests that the negative effect of E427 at position −4 of ZnF4 when juxtaposed to 5caC is about the same as losing binding by ZnF4 altogether. It also suggests that Frasier syndrome stems from perturbed binding at genomic sites that contain C or 5mC (by the increased amount of −KTS isoform) rather than at sites containing 5caC (owing to similar binding affinities for both isoforms). We found that the +KTS isoform with Q369 at position −4 binds most strongly to 5caC-containing DNA with decreasing affinity in the order 5caC > 5mC ≈ C > 5hmC ≈ 5fC (Fig. 4D) and that the affinity increases markedly at lower salt concentrations (Fig. 4E).

### A WT1 mutant variant with high preference for 5mC

**Figure 5.**
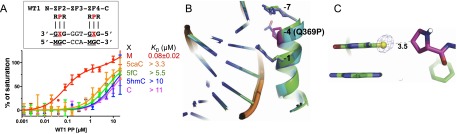
A WT1 mutant variant with high preference for 5mC. (*A*) The “PP” variant (Q369P and E427P) of WT1 prefers 5mC to all other forms by a factor of 40—140. (*B*) Structural comparison of WT1 wild type and the Q369P mutant. (*C*) The methyl group of 5mC forms a van der Waals contact with the proline at −4 position. The simulated annealing omit electron density (blue lines), contoured at 4σ above the mean, for omitting the methyl group of 5mC are shown.

Finally, we were interested in variants that strongly distinguish only one modification. The engineered QQ variant of WT1 strongly distinguishes sequences containing 5caC, 5mC, or C from those containing 5hmC or 5fC by a factor of 60 or more (Fig. 1I). Another variant that we examined (“PP”) has proline at position −4 in WT1 ZnF2 and ZnF4 in place of Q369 and E427. This variant displayed a high preference for 5mC compared with both oxidized C and unmodified C by factors ranging from 40 to 140 (Fig. 5A). This result was somewhat surprising. We anticipated that proline in the first turn (the third residue) of the helices might destabilize them (Stoll et al. 2007) and abolish binding, but, evidently, they did not (Fig. 5B,C). The selectivity of the PP WT1 variant for 5mC compared with C stems not from an increase in affinity for 5mC but a >100-fold decrease in affinity for C (cf. Figs. 5A and 1D). Interestingly, the mismatch repair endonuclease MutH (Lee et al. 2005) also uses a proline to juxtapose a methyl group in its hemimethylated recognition sequence, one that occurs on adenine rather than on C in this case (Supplemental Fig. S5).

## Discussion

Our results show that the binding domains of transcription factors Egr1 and WT1 are responsive to all forms of modified C within the recognition sequence. They display high affinity for sequences containing C and slightly higher affinity for sequences containing 5mC but much lower affinity for the sequences containing 5hmC or 5fC. The two domains distinguish primarily unoxidized forms of C from oxidized forms, rather than the more familiar situation of unmethylated C from methylated C.

The most interesting aspect of our study concerns the 5caC modification. Put simply, when residue −4 is E, sequences containing 5caC are bound the poorest, and when it is Q, they are bound the best. When E juxtaposes 5caC, both carboxylate groups bear a full −1 charge and thus repel one another electrostatically. We consider this the likeliest explanation for the very poor binding affinity of E:5caC combinations. In contrast, when Q juxtaposes 5caC, a strong H-bond forms between the carboxylate and side chain amide groups, one that is likely augmented by electrostatic attraction. 5caC has the potential to function epigenetically to weaken binding by ZnFs with E at position −4 and strengthen binding by ZnFs with Q at position −4.

Our analyses show that binding by Egr1 and WT1 is affected to different degrees depending on whether one or both sites are modified. Although the experiments were performed in vitro, they imply that ZnF transcription factors can respond in modulated ways to alternative modifications at different C positions. Because the Egr1–WT1-binding sequence has two CpG sites, in principle, it can occur in 25 different states, with C, 5mC, 5hmC, 5fC, or 5caC at either site. If the two DNA strands can be modified independently (i.e., strand-biased DNA modification) (Yu et al. 2012), then the number of different states could swell to 25^2^ (or 625). Many of these states could affect binding affinity, and so gene activity could plausibly be controlled on a much finer scale by these modifications than simply “on” or “off.” This hints, perhaps, at new levels of subtlety and versatility in epigenetic regulatory processes. In the case of WT1, the splice variants add yet another layer of regulatory control (Fig. 4).

## Materials and methods

Rather than following published methods of refolding insoluble WT1 ZnF1–4 (Laity et al. 2000a) and Egr1/Zif268 (Pavletich and Pabo 1991), we expressed and purified these proteins in soluble form by fusing the three-ZnF DNA-binding domains to glutathione S-transferase (GST). Because the published X-ray structure of WT1 has only 3.15 Å resolution (Stoll et al. 2007), which is insufficient to discern the various C modifications, we sought a higher-resolution structure. We purified a construct comprising WT1 ZnF2–4 and determined its structure anew in complex with oligos containing various C modifications to final resolutions between 1.5 and 2.1 Å.

### Protein expression and purification

GST-tagged human Egr1/Zif268 residues 335–423 (NP_001955.1) and human WT1 residues 401–488 (NP_000369.3; −KTS isoform) were separately cloned into pGEX6P-1, generating plasmids of pXC1272 and pXC1295. In addition, we generated two WT1 mutants (QQ variant, pXC1335; PP variant, pXC1320) and the +KTS isoform (pXC1329). These were expressed in the *Escherichia coli* strain of BL21-CodonPlus(DE3)-RIL (Stratagene). Typically, 2–3 L of cultures were grown at 37°C to log phase (OD_600_ ∼ 0.5–0.8) and then shifted to 16°C, ZnCl_2_ was added to a final concentration of 25 μM, expression was induced by the addition of β-D-1-thiogalactopyranoside to 0.2 mM, and the cultures were incubated overnight at 16°C. Cells were harvested by centrifugation; resuspended in lysis buffer containing 20 mM Tris-HCl (pH 7.5), 250 mM NaCl (Egr1/Zif268) or 500 mM NaCl (WT1), 5% (v/v) glycerol, 0.5 mM Tris(2-carboxyethyl)phosphine hydrochloride (TCEP), and 25 μM ZnCl_2_; and lysed by sonication. Lysates were mixed with polyethylenimine (Sigma) at pH 7.0 (adjusted by NaOH) to a final concentration of 0.4% (w/v) before centrifugation at 18,000 rpm.

The cleared extract was loaded onto a glutathione-Sepharose 4B column (GE Healthcare) pre-equilibrated with lysis buffer (above). The GST fusion proteins were eluted with 20 mM glutathione (GSH) in the elution buffer containing 100 mM Tris-HCl (pH 8.0), 5% (v/v) glycerol, 25 μM ZnCl_2_, and 250 mM NaCl (Egr1/Zif268) or 500 mM NaCl (WT1). The GST tag was removed using PreScission protease (purified in-house), leaving five additional N-terminal residues (Gly–Pro–Leu–Gly–Ser) on each protein. The proteins were diluted twofold with 20 mM Tris-HCl (pH 7.5), 5% (v/v) glycerol, 25 μM ZnCl_2_, and 0.5 mM TCEP and loaded onto tandem HiTrap-Q/HiTrap-SP columns (GE Healthcare). Most proteins flowed through the Q column onto the SP column from which it was eluted using a linear gradient of NaCl from 120 mM to 1 M. Finally, the pooled protein was concentrated and loaded onto a size exclusion column and eluted as a single peak in 500 mM NaCl, 20 mM Tris-HCl (pH 7.5), 5% (v/v) glycerol, and 25 μM ZnCl_2_. Final protein concentrations were estimated by absorbance at 280 nm for WT1 (absorbance coefficient of 9.66 for 1 mM WT1) or, for Egr1/Zif268, by Bradford protein assay (Bio-Rad no. 500-0205) using a mutant Zfp57 E182Y (Liu et al. 2013a) as a standard.

### Fluorescence-based DNA-binding assay

Fluorescence polarization measurements were carried out at 25°C on a Synergy 4 microplate reader (BioTek). The 6-carboxy-fluorescein (FAM)-labeled dsDNA probe (5 nM) was incubated for 10 min with increasing amounts of protein in 300 mM NaCl, 20 mM Tris-HCl (pH 7.5), 5% (v/v) glycerol, and 0.5 mM TCEP. No change in fluorescence intensity was observed with the addition of protein. The sequences of the oligonucleotides were FAM-5′-TAYGCCCAYGC-3′ and 3′-TGXGGGTGXGA-5′ (where X and Y = C, 5mC, 5hmC, 5fC, or 5caC as defined in Fig. 1). Curves were fit individually using GraphPad Prism 5.0 software (GraphPad Software, Inc.). Binding constants (*K*_D_) were calculated as [*mP*] = [maximum *mP*] × [*C*]/(*K*_D_ + [*C*]) + [baseline *mP*], and saturated [*mP*] was calculated as saturation = ([*mP*] − [baseline *mP*])/([maximum *mP*] − [baseline *mP*]), where *mP* is millipolarization and [*C*] is protein concentration. Averaged *K*_D_ and its standard error are reported.

### Crystallography

We crystallized Egr1/Zif268 (or WT1) in the presence of DNA by the sitting-drop vapor diffusion method at 16°C using equal amounts of protein–DNA mixtures (1 mM) and well solution (Supplemental Table S2). Protein–DNA mixtures in equimolar ratios were incubated for 30 min at 16°C before crystallization. Crystals were cryoprotected by soaking in mother liquor supplemented with 20% (v/v) ethylene glycol or 20% (v/v) glycerol before plunging into liquid nitrogen.

X-ray diffraction data sets were collected at 100K at the SER-CAT beamlines (22BM-E and 22ID-D) at the Advanced Photon Source, Argonne National Laboratory, and processed using HKL2000 (Otwinowski et al. 2003). Initial crystallographic phases were determined by molecular replacement using the coordinates of the DNA-binding domains of human Egr1/Zif268 (Protein Data Bank [PDB]: 1AAY) (Elrod-Erickson et al. 1996) and WT1 (PDB: 2PRT) (after deleting ZnF1) (Stoll et al. 2007) as search models, respectively (Supplemental Figs. S2, S3). Phasing, molecular replacement, map production, and model refinement were performed using PHENIX (Adams et al. 2010). All eight structures were solved, built, and refined independently. The statistics were calculated for the entire resolution range (Supplemental Table S1). The *R*_free_ and *R*_work_ values were calculated for 5% (randomly selected) and 95%, respectively, of the observed reflections. Molecular graphics were generated using PyMol (DeLano Scientific, LLC).

### Accession numbers

The X-ray structures (coordinates and structure factor files) of Egr1/Zif268 (4R2A for 5mC, 4R2C for 5hmC, and 4R2D for 5fC), WT1 (4R2E for 5mC, 4R2P for 5hmC, 4R2Q for 5fC, and 4R2R for 5caC), and WT1 mutant Q369P in complex with 5mC DNA (4R2S) have been submitted to PDB.

## Supplementary Material

Supplemental Material
