# Supplementary material for: Wilms tumor protein recognizes 5-carboxylcytosine within a specific DNA sequence
Source: Genes Dev. 2014 Oct 15;28(20):2304–13. doi: 10.1101/gad.250746.114 (PMC4201290; doi:10.1101/gad.250746.114)
Supplement: Supplemental Material [file supp_28_20_2304__index.html]

Wilms tumor protein recognizes 5-carboxylcytosine within a specific DNA sequence — Supplemental Material 

# Wilms tumor protein recognizes 5-carboxylcytosine within a specific DNA sequence

## Supplemental Material

**Files in this Data Supplement:**

- Supplemental Material.pdf
