# Supplementary material for: Wilms tumor protein recognizes 5-carboxylcytosine within a specific DNA sequence
Source: Genes Dev. 2014 Oct 15;28(20):2304–13. doi: 10.1101/gad.250746.114 (PMC4201290; doi:10.1101/gad.250746.114)
Supplement: Supplemental Material [file supp_gad.250746.114_Supplemental_Material.pdf]

Supplemental Material

Wilms tumor protein recognizes 5-carboxylcytosine within a specific DNA sequence

Hideharu Hashimoto[1], Yusuf Olatunde Olanrewaju[1], Yu Zheng[2], Geoffrey G. Wilson[2], Xing Zhang[1], and Xiaodong Cheng[1,*]

[1] Department of Biochemistry, Emory University School of Medicine, Atlanta, GA 30322, USA

[2] New England Biolabs, 240 County Road, Ipswich, MA 01938, USA

Corresponding author: xcheng@emory.edu

Email addresses for all authors:

HH (hhashi3@emory.edu)

YOO (yusuf.o.olanrewaju@emory.edu)

YZ (zhengy@neb.com)

GGW (wilson@neb.com)

XZ (xzhan02@emory.edu)

XC (xcheng@emory.edu)

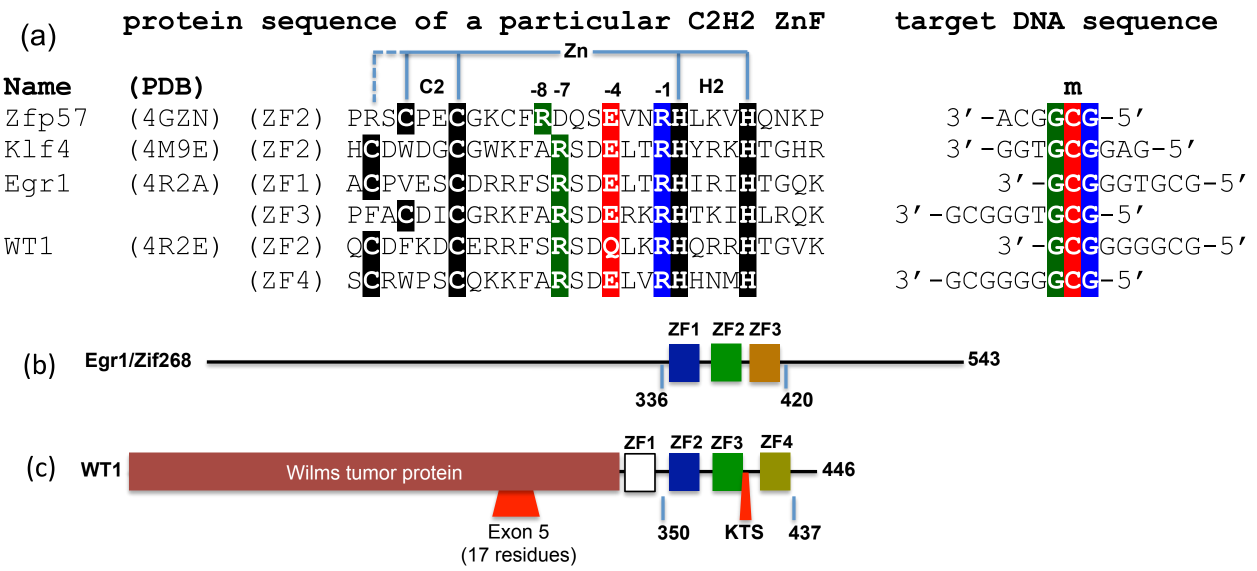

Supplemental Figure S1. Schematic representation of human Egr1/Zif268 and human WT1. (**a**) Sequence alignment of representative C2H2 ZnF recognizing the DNA sequence GCG. The protein sequences are printed N-to-C, and the DNA sequences, 3'-to-5'. The amino acids at positions -1, -4 and -7 (or -8) are highlighted with the same color codes (blue, red, and green) as the bases they recognize. "m" indicates the position of the cytosine that is modified. Two cysteine and two histidine residues (C2H2) in each finger are responsible for Zn^{2+} ligand binding (upper connecting lines), coordinate a zinc ion in tetrahedral geometry. This C2H2-Zn^{2+} cluster is located on the outer surface of the protein and does not participate in sequence-recognition. (**b**) Human Egr1/Zif268 contains a C-terminal ZnF DNA binding domain comprising three fingers in tandem. (**c**) Human WT1 contains a C-terminal ZnF DNA binding domain comprising four fingers in tandem. *WT1* is a complex gene characterized by many isoforms (~36) (Ozdemir and Hohenstein, 2014). The four most studied isoforms differ in the presence or absence of exon 5 and KTS. For the study described here, we used a fragment of WT1 containing ZnF2, 3 and 4 without KTS (the –KTS isoform), that being the major DNA-binding isoform (Ozdemir and Hohenstein, 2014), and with KTS (the +KTS isoform, see Fig. 4).

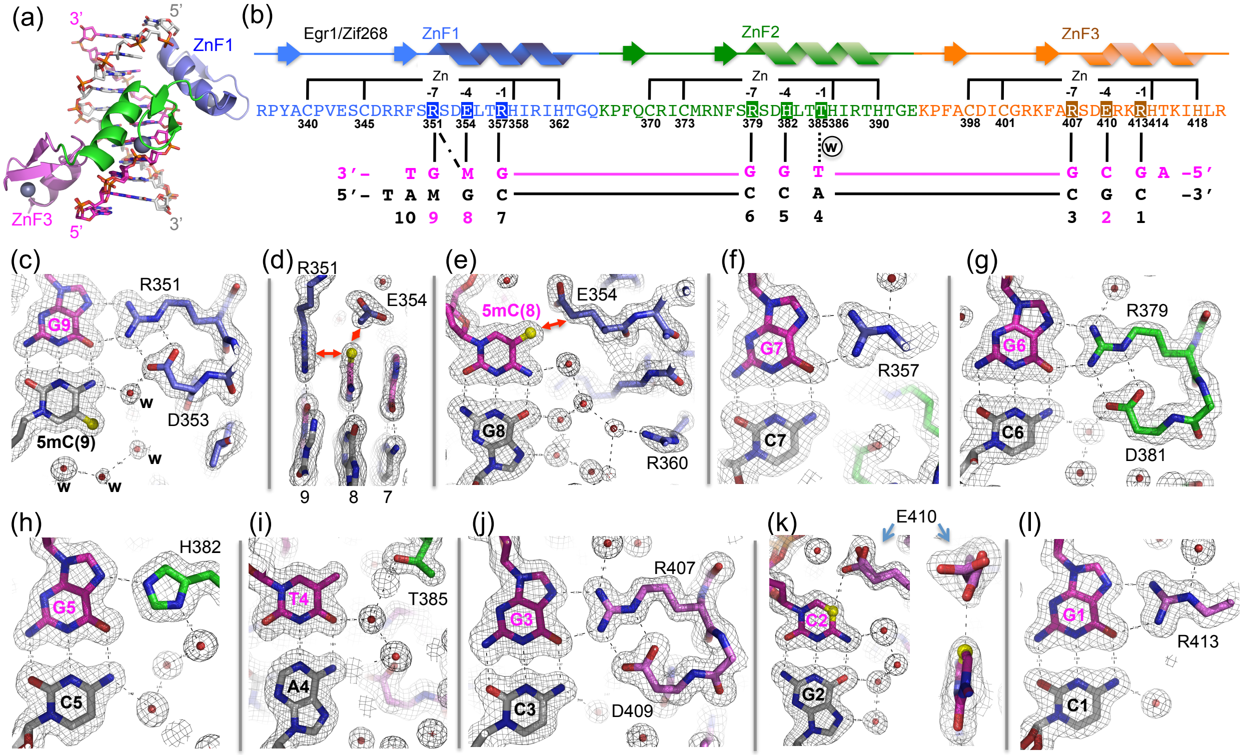

Supplemental Figure S2. Structure of Egr1/Zif268 bound with methylated DNA. (**a**) The human Egr1 ZnF protein binds in the major groove of DNA with ZnF1 (blue), ZnF2 (green) and ZnF3 (pink). Base-specific contacts involve primarily one DNA strand (magenta), the 3'-to-5' orientation of which coincides with the N-to-C orientation of the protein. (**b**) Schematic representation of the ZnF DNA-binding domain of Egr1. Arrows represent β-strands; lines, loops; and ribbons, α-helices. Amino acids at positions -1, -4, and -7 (highlighted) relative to the first histidine interact specifically with the DNA bases. 'M' indicates the methylated cytosine. (**c**) R351 at ZnF1 position -7 forms H-bonds (dotted lines) with Gua9 (*O*6 and *N*7). D353 at position -5 stabilizes R351 through H-bonds. It also forms a water-mediated H-bond with 5mC (*N*4) in the bottom strand, the methyl group of which is surrounded by ordered water molecules ('w'). (**d**) The top strand 5mCpG interacts with R351 and forms a 5mC-Arg-Gua triad. (**e**) The Cγ atom of E354 forms a van der Waals interaction with the methyl group of upper strand 5mC. A network of water molecules is present at the protein-DNA interface. (**f**) R357 forms two H-bonds with Gua7 (*O*6 and *N*7). (**g**) The interactions of R379 and D381 at position -7 and -5 of ZnF2 are similar to those at base pair 9 (panel c). (**h**) H382 forms one H-bond with Gua5 (*N*7). (**i**) T385 interacts with Thy4 via water molecules. The DNA sequence in this position is variable and can accommodate guanine instead. (**j**) The interactions of R407 and D409 at position -7 and -5 of ZnF3 are similar to those at base pairs 9 and 6 (panels c and g). (**k**) E410 at -4 position adopts two conformations, forming a van der Waals interaction or a weak C-H…O type of hydrogen bond with the ring C5 atom of unmodified Cyt2. (**l**) The R413-Gua1 interaction of ZnF3 is similar to the R357-Gua7 interaction of ZnF1 (panel f).

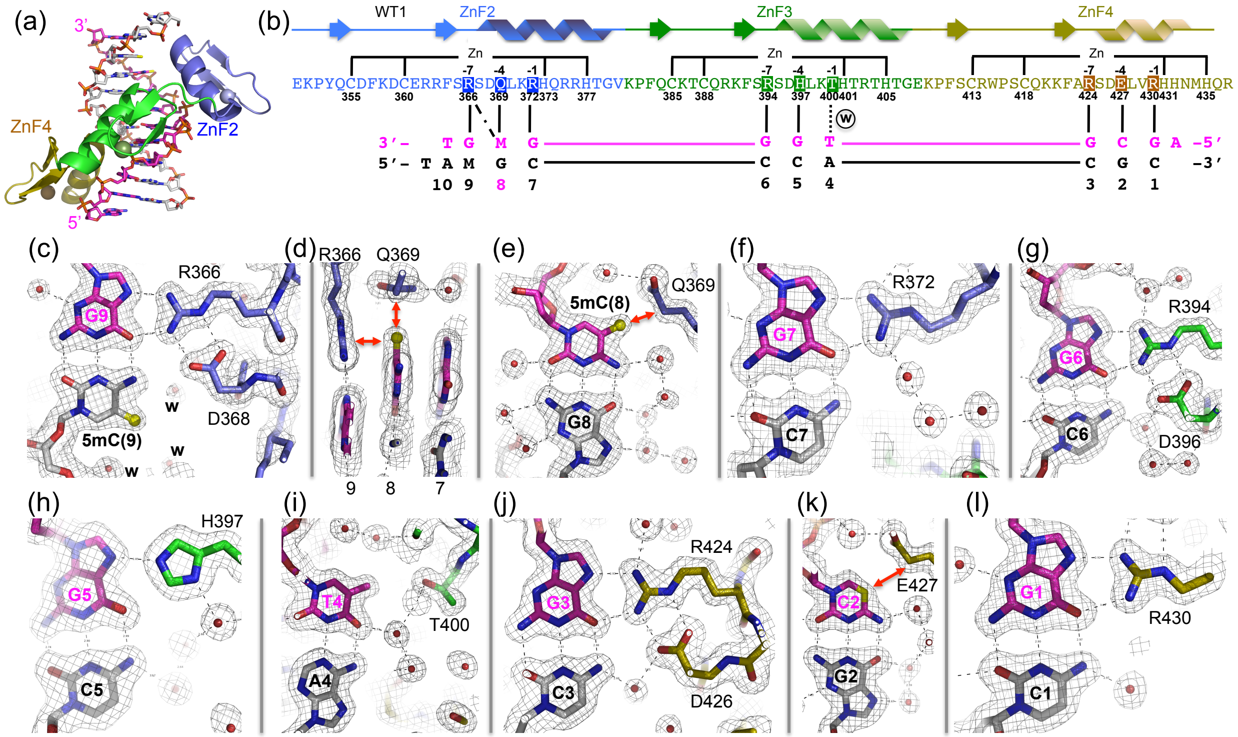

Supplemental Figure S3. Structure of WT1 bound with methylated DNA. (**a**) The human WT1 ZnF protein (ZnF2-4) binds in the major groove of DNA with ZnF2 (blue), ZnF3 (green) and ZnF4 (brown). (**b**) Schematic representation of human WT1 DNA-binding ZnF domain. (**c**) R366 and D368 at positions -7 and -5 of ZnF2 interactions with Gua9. (**d**) The top strand 5mCpG interacts with R366 and forms a 5mC-Arg-Gua triad. (**e**) The carbon Cγ atom of Q369 forms a hydrophobic interaction with the methyl group of the top strand 5mC. Ordered water molecules interact from both major (right) and minor (left) grooves. (**f**) R372-Gua7 interaction. (**g**) R394 at -7 and D396 at -5 positions of ZnF3 interact with Gua6. (**h**) H382-Gua5 interaction. (**i**) T400 involves in water-mediated interactions with Thy4. The DNA sequence in this position is variable and can also accommodate guanine. (**j**) R424 at -7 and D426 at -5 positions of ZnF4 interaction with Gua3. (**k**) E427 at -4 position forms a van der Waals contact with the ring C5 atom of unmodified Cyt2. (**l**) R430-Gua1 interaction.

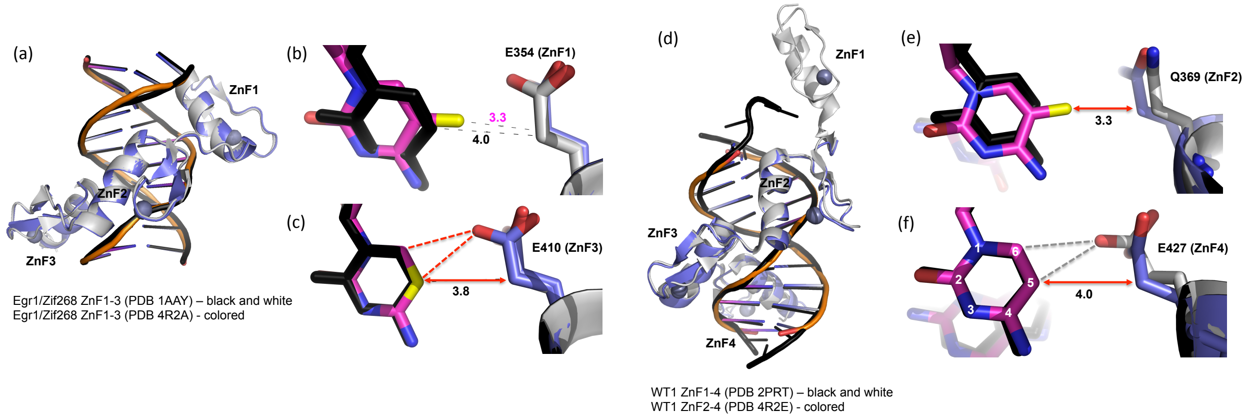

Supplemental Figure S4. Structural comparison of methylated and unmethylated DNA complexes with Egr1/Zif268 and WT1. (**a**) Superimposition of Egr1/Zif268 with unmethylated DNA (pdb:1AAY) and methylated DNA (this study). (**b**) Methylation in the 3' GCG reduces the separation between E354 and the base, from 4.0Å with C (black) to 3.3Å with 5mC. (**c**) The side chain of E410 adopts two conformations much like E427 of WT1 (see panel f). One might interact with the base through van der Waals contact, the other through C-H…O type H-bonds. It is interesting to note that whereas glutamate (E) juxtaposes cytosine (and 5mC in this study) in Egr1/Zif268 naturally (Pavletich and Pabo, 1991), aspartate (D) was found to preferentially juxtapose it in a phage display selection study (Choo and Klug, 1994). This finding led Choo and Klug to comment that "The physical basis for the interaction of aspartate/glutamate and cytosine is not yet clear, since hydrogen-bonding contacts between these groups have yet to be observed in zinc finger co-crystal structures" (Choo and Klug, 1997). In more recent bacterial one-hybrid experiments where only unmodified bases were present, aspartate at position -4 was again found to preferentially juxtapose cytosine as the middle base of the recognition triplet (Gupta et al., 2014). In addition, proline at position -4 was found to preferentially juxtapose thymine presumably through interaction with the thymine 5-methyl group (Gupta et al., 2014). (**d**) Superimposition of WT1 ZnF1-4 (pdb:2PRT) and WT1 ZnF2-4 (this study). ZnF2-4 recognize the 9-bp consensus sequence; ZnF1 is superfluous. (**e**) Methylation in the cytosine in the 3' GCG triplet in the consensus sequence brings the side-chain of Q369 within van der Waals contact (3.3Å) of the 5mC. Q369 and the unmethylated cytosine at this position (black) in pdb:2PRT are farther apart. (**f**) The unmethylated cytosine in the 5' GCG triplet in our structure superimposes well on that in pdb:2PRT (black). The side chain of E427 adopts two conformations, one in van der Waals contact with the ring C5 carbon atom (4Å; this study), the other with weak C-H…O type H-bonds with the ring atoms C5 and C6 (pdb:2PRT) (much like E410 of Egr1 as panel c).

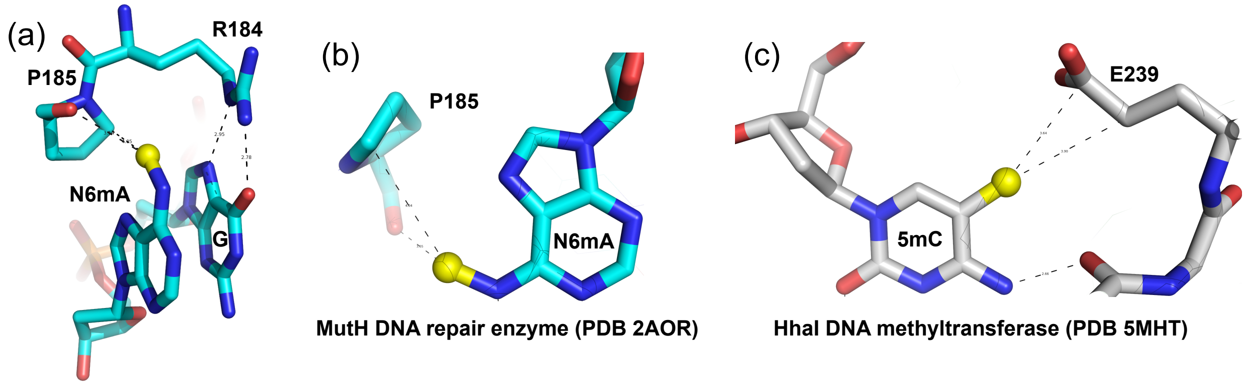

Supplemental Figure S5. Examples of amino acid interactions with methylated bases. (**a**) MutH nicking endonuclease recognizes G**A**TC and binds preferentially when one strand contains A and the other contains N6-methyladenine (N6mA) (Welsh et al., 1987). A methyl-Arg-Gua triad occurs in the binding site (Lee et al., 2005), much as in Egr1 and WT1. (**b**) When bound to the hemimethylated sequence, proline (Pro184) juxtaposes N6mA in perpendicular configuration (pdb:2AOR), confirming the appropriateness of this kind of interaction. (**c**) HhaI methyltransferase recognizes G**C**GC, and binds preferentially when one strand contains C and the other 5mC (underlined) (O'Gara et al., 1996). When bound to the hemimethylated sequence, glutamate (E239) juxtaposes 5mC in perpendicular configuration (pdb:5MHT), much as E354 of Egr1 and Q369 of WT1 do at the GCG ZnF triplet binding sequence.

Supplemental Text

To learn more about the functions of modified cytosines in mammalian genomes, and about the mechanisms that control their locations, identities, and levels, methods are needed to distinguish the modifications individually, and to map their positions accurately. Quantitative single-base resolution sequencing of 5mC (Lister et al., 2009), 5hmC (Booth et al., 2012; Sun et al., 2013; Yu et al., 2012), and more recently 5fC (Booth et al., 2014; Song et al., 2013) are available, but none for 5caC.

In one study using modification-specific antibodies (Shen et al., 2013), genome-wide maps of regions containing 5fC and 5caC were generated for wild-type mouse embryonic stem cells (mESC) and for thymine DNA glycosylase (TDG)-deficient mESC cells in which 5fC and 5caC accumulates(He et al., 2011). Using this dataset, we examined the co-incidence of Egr1/WT1 binding sequences and regions containing 5caC and 5fC. No binding sequences were found in the 5caC-containing regions of wild-type mESC (0 out of 1673), but a substantial number (827 out of 89,503 5caC-containing regions) were found in TDG-deficient mESC. Similar results were obtained for 5fC: 31 out of 24,482 5fC-containing regions in wild-type, and 316 out of 45,360 in TDG-deficient mESC. In a separate study of 5fC in mESC (Booth et al., 2014), we found only two positions at which 5fC and the Egr1/WT1 sequence overlap. These observations suggest that Egr1/WT1 binding sites are being dynamically modified in mESC, and that 5fC and 5caC are being actively removed by TDG (He et al., 2011).

References

Booth MJ, Branco MR, Ficz G, Oxley D, Krueger F, Reik W, Balasubramanian S. 2012. Quantitative sequencing of 5-methylcytosine and 5-hydroxymethylcytosine at single-base resolution. *Science* **336**: 934-937.

Booth MJ, Marsico G, Bachman M, Beraldi D, Balasubramanian S. 2014. Quantitative sequencing of 5-formylcytosine in DNA at single-base resolution. *Nature Chemistry* **6**: 435-440.

Choo Y, Klug A. 1994. Selection of DNA binding sites for zinc fingers using rationally randomized DNA reveals coded interactions. *Proc Natl Acad Sci U S A* **91**: 11168-11172.

Choo Y, Klug A. 1997. Physical basis of a protein-DNA recognition code. Curr Opin Struct Biol *7*, 117-125.

Gupta A, Christensen RG, Bell HA, Goodwin M, Patel RY, Pandey M, Enuameh MS, Rayla AL, Zhu C, Thibodeau-Beganny S, Brodsky MH, Joung JK, Wolfe SA, Stormo GD. 2014. An improved predictive recognition model for Cys2-His2 zinc finger proteins. *Nucleic Acids Res* **42**: 4800-4812.

He YF, Li BZ, Li Z, Liu P, Wang Y, Tang Q, Ding J, Jia Y, Chen Z, Li L, Sun Y, Li X, Dai Q, Song CX, Zhang K, He C, Xu GL. 2011. Tet-mediated formation of 5-carboxylcytosine and its excision by TDG in mammalian DNA. *Science* **333**: 1303-1307.

Lee JY, Chang J, Joseph N, Ghirlando R, Rao DN, Yang W. 2005. MutH complexed with hemi- and unmethylated DNAs: coupling base recognition and DNA cleavage. *Mol Cell* **20**: 155-166.

Lister R, Pelizzola M, Dowen RH, Hawkins RD, Hon G, Tonti-Filippini J, Nery JR, Lee L, Ye Z, Ngo QM, Edsall L, Antosiewicz-Bourget J, Stewart R, Ruotti V, Millar AH, Thomson JA, Ren B, Ecker JR. 2009. Human DNA methylomes at base resolution show widespread epigenomic differences. *Nature* **462**: 315-322.

O'Gara M, Roberts RJ, Cheng X. 1996. A structural basis for the preferential binding of hemimethylated DNA by HhaI DNA methyltransferase. *J Mol Biol* **263**: 597-606.

Ozdemir DD, Hohenstein P. 2014. Wt1 in the kidney--a tale in mouse models. *Pediatric Nephrology* **29**: 687-693.

Pavletich NP, Pabo CO. 1991. Zinc finger-DNA recognition: crystal structure of a Zif268-DNA complex at 2.1 A. *Science* **252**: 809-817.

Shen L, Wu H, Diep D, Yamaguchi S, D'Alessio AC, Fung HL, Zhang K, Zhang Y. 2013. Genome-wide analysis reveals TET- and TDG-dependent 5-methylcytosine oxidation dynamics. *Cell* **153**: 692-706.

Song CX, Szulwach KE, Dai Q, Fu Y, Mao SQ, Lin L, Street C, Li Y, Poidevin M, Wu H, Gao J, Liu P, Li L, Xu GL, Jin P, He C. 2013. Genome-wide profiling of 5-formylcytosine reveals its roles in epigenetic priming. *Cell* **153**: 678-691.

Sun Z, Terragni J, Borgaro JG, Liu Y, Yu L, Guan S, Wang H, Sun D, Cheng X, Zhu Z, Pradhan S, Zheng Y. 2013. High-resolution enzymatic mapping of genomic 5-hydroxymethylcytosine in mouse embryonic stem cells. *Cell Reports* **3**: 567-576.

Welsh KM, Lu AL, Clark S, Modrich P. 1987. Isolation and characterization of the Escherichia coli mutH gene product. *J Biol Chem* **262**: 15624-15629.

Yu M, Hon GC, Szulwach KE, Song CX, Zhang L, Kim A, Li X, Dai Q, Shen Y, Park B, Min JH, Jin P, Ren B, He C. 2012. Base-resolution analysis of 5-hydroxymethylcytosine in the Mammalian genome. *Cell* **149**: 1368-1380.

Supplemental Table S1. Statistics of X-ray diffraction and Refinement

Protein	Egr1/Zif268			WT1				WT1 (Q369P)
DNA	5mC/5mC	5hmC/5hmC	5fC/5fC	5mC/5mC	5hmC/5hmC	5fC/5fC	5caC/5mC	5mC/5mC
PDB	4R2A	4R2C	4R2D	4R2E	4R2P	4R2Q	4R2R	4R2S
Space Group	$C222_1$	$C222_1$	$C222_1$	$P2_12_12$	$P2_12_12$	$P2_12_12$	$P2_12_12$	C2
Unit cell (a, b, c (Å))	44.0, 56.2, 130.2	44.0, 56.1, 129.6	44.0, 55.9, 127.9	67.1, 78.3, 35.6	66.9, 78.6, 35.7	67.1, 78.7, 35.7	67.0, 77.5, 35.6	69.1, 66.6, 36.1
α, β, γ (°)	90, 90, 90	90, 90, 90	90, 90, 90	90, 90, 90	90, 90, 90	90 90, 90	90, 90, 90	90, 93.3, 90
Resolution (Å) *	28.10-1.59 (1.65-1.59)	28.04-1.89 (1.96-1.89)	34.55-2.09 (2.16-2.09)	29.17-1.84 (1.91-1.84)	26.49-1.79 (1.85-1.79)	19.4-1.54 (1.60-1.54)	29.1-2.09 (2.16-2.09)	29.4-2.49 (2.58-2.49)
[a] R_{merge} *	0.053 (0.386)	0.072 (0.463)	0.057 (0.347)	0.066 (0.381)	0.067 (0.428)	0.078 (0.668)	0.079 (0.427)	0.087 (0.250)
[b] <I/σI> *	28.9 (3.6)	29.9 (4.6)	38.3 (7.4)	23.1 (3.5)	27.3 (3.8)	17.7 (2.2)	13.1 (3.5)	19.9 (6.6)
Completeness (%) *	99.1 (95.8)	100.0 (100.0)	98.1 (96.7)	99.3 (94.2)	99.0 (92.9)	98.5 (99.9)	99.8 (98.7)	98.5 (92.1)
Redundancy *	7.3 (5.6)	10.0 (8.6)	12.8 (12.3)	6.0 (4.7)	8.5 (6.4)	5.6 (4.6)	8.1 (5.1)	7.3 (6.6)
Obs. Reflections	160,131	132,833	121,667	100,710	154,979	158,030	92,469	41,317
Unique reflections *	21,981 (2098)	13,248 (1279)	9,534 (893)	16,827 (1564)	18,227 (1669)	28,341 (2798)	11,476 (1090)	5690 (545)
**Refinement**								
Resolution (Å)	1.59	1.89	2.09	1.84	1.79	1.54	2.09	2.49
No. Reflections	21,919	13,207	9,497	15,950	18,240	28,304	11,418	5645
[c] R_{work}/ [d] R_{free}	13.7/18.9	19.9/25.0	19.8/24.0	16.2/20.8	17.6/20.8	16.1/19.2	19.3/22.3	20.8/26.8
No. Atoms								
Protein	722	718	718	747	759	770	745	711
DNA	497	514	520	488	562	544	493	488
Zn	3	3	3	3	3	3	3	3
Solvent	193	185	83	149	160	184	116	33
B-factors (Å)								
Protein	28.0	29.8	35.7	24.7	24.9	25.0	29.2	36.9
DNA	25.5	28.0	36.4	24.3	21.9	25.0	28.0	34.8
Zn	23.9	25.1	27.8	23.6	21.2	20.9	26.6	40.2
Solvent	34.3	33.0	36.4	32.3	30.4	33.6	31.6	35.4
R.m.s. deviations								
Bond length (Å)	0.012	0.009	0.004	0.012	0.014	0.007	0.004	0.004
Bond angles (°)	1.5	0.8	0.6	1.4	1.7	1.1	0.7	0.7
All atom clash score	3.6	1.4	3.1	2.7	5.9	3.3	0.9	0.9
Ramachandran Plot (%)	100	99	100	100	99	100	99	100
Additional Allowed	0	1	0	0	1	0	1	0
C_β deviation	0	0	0	0	0	0	0	0

Note: Wavelength=1 Å; one complexes per asymmetric unit; Synchrotron beamline APS 22-BM or 22-ID

*Values in parenthesis correspond to highest resolution shell; [a] $R_{merge} = \Sigma \mid I - <I> \mid / \Sigma I$, where I is the observed intensity and <I> is the averaged intensity from multiple observations; [b] <I/σI> = averaged ratio of the intensity (I) to the error of the intensity (σI); [c] $R_{work} = \Sigma \mid F_{obs} - F_{cal} \mid / \Sigma \mid F_{obs} \mid$, where F_{obs} and F_{cal} are the observed and calculated structure factors, respectively; [d] R_{free} was calculated using a randomly chosen subset (5%) of the reflections not used in refinement.

Supplemental Table S2. Summary of crystallization conditions

Protein	DNA	Conditions
Egr1/Zif268	5mC/5mC	12% (w/v) PEG 3350, 4% (v/v) Tacsimate
	5hmC/5hmC	35% (v/v) (5/4 PO/OH), 0.2 M KCl, 50 mM Hepes-NaOH pH 7.5
	5fC/5fC	30% (w/v) PEG 400, 0.1 M CHES pH 10.5
WT1	5mC/5mC	10% (w/v) PEG 6000, 5% MPD, 0.1 M HEPES-NaOH pH 7.5
	5hmC/5hmC	20% (w/v) PEG 10,000, 0.1 M HEPES-NaOH, pH 7.5
	5fC/5fC	12% (w/v) PEG 3350, 4% (v/v) Tacsimate
	5caC/5mC	28% (w/v) PEGMME 2000, 0.1 M BisTris pH 6.5
WT1 (Q369P)	5mC/5mC	12% (w/v) PEG 3350, 0.1 M Ammonium Citrate

Note: Temperature = 16 °C;
PEG = polyethylene glycol;
PEGMME = polyethylene glycol monomethyl ether;
Tacsimate = a mixture of titrated organic acid salts (Hampton Research);
(5/4 PO/OH) = pentaerythritol propoxylate (Hampton Research)
